# Draft Genome Sequence of *Lactobacillus helveticus* ATCC 12046

**DOI:** 10.1128/genomeA.01595-17

**Published:** 2018-02-15

**Authors:** María Mercedes Palomino, Germán F. Burguener, Josefina Campos, Mariana Allievi, Joaquina Fina-Martin, Mariano Prado Acosta, Darío A. Fernández Do Porto, Sandra M. Ruzal

**Affiliations:** a Departamento de Química Biológica, Pabellón 2, Ciudad Universitaria, Facultad de Ciencias Exactas y Naturales, UBA, IQUIBICEN/CONICET, Buenos Aires, Argentina; b Instituto de Cálculo, Pabellón 2, Ciudad Universitaria, Facultad de Ciencias Exactas y Naturales, UBA, Buenos Aires, Argentina; c Instituto Dr. Carlos G. Malbrán, Buenos Aires, Argentina

## Abstract

*Lactobacillus helveticus* is a lactic acid bacterium used traditionally in the dairy industry, especially in the manufacture of cheeses. We present here the 2,141,841-bp draft genome sequence of *L. helveticus* strain ATCC 12046, a potential starter strain for improving cheese production.

## GENOME ANNOUNCEMENT

*Lactobacillus helveticus* is an industrial thermophilic starter or adjunct culture, predominantly used in the fermentation of milk and the manufacture of cheeses (1). Some studies have underlined the health-promoting potential of many strains of *L. helveticus*, mediated by surface protein from their cell envelope (2–6).

*L. helveticus* ATCC 12046 was obtained from the Centro de Referencia para Lactobacilos (CERELA, Tucumán, Argentina). The genome sequence was obtained using a whole-genome shotgun strategy with Illumina MiSeq technology. The number of paired-end reads was approximately 402,173 in pairs of 251 bp, totaling 192 Mb of sequence data. *De novo* assembly of the contig was done using SPAdes version 3.9.0 (7) and SSPACE version 3.0 (8) and later by means of the reference genome to reduce fragmentation. This assembly generated 64 scaffolds, with an *N*_50_ value of 89,074 bp and an *L*_50_ value of 9 bp. After *de novo* assembly, contigs were sorted against *L. helveticus* CNRZ 32 since 92.63% of the sequenced reads were mapped to its genome (with 93.7% coverage). The draft genome is 2,141,841 bp in length. The G+C content is 36.5 mol%. Genome annotation was done using the standard operating procedures from our own prokaryotic annotation pipeline based on Glimmer for open reading frame prediction (9). Functional annotation included protein function, Gene Ontology (GO) and Clusters of Orthologous Groups (COG) terms, Enzyme Commission (EC) numbers, and Pfam database domains. A total of 2,305 coding sequences (CDSs) and 66 structural RNAs (61 tRNAs) were predicted; 582 CDSs (25%) were classified as hypothetical or uncharacterized proteins. The Rapid Annotations using Subsystems Technology (RAST) server (10) was used for subsystem descriptions. According to RAST, the annotation assigned 923 CDSs (40%) to RAST subsystems.

In common with *L. helveticus* DPC 4571, the genome presents a large number of insertion sequences (ISs) (11). It includes 201 mobile element proteins with high similarity to transposase enzyme genes, indicating that they may belong to IS elements, which have been shown to speciﬁcally contribute to niche adaptation by promoting a variety of genetic rearrangements. In comparison with CNRZ 32, the genome presents four deleted regions ranging from 10 to 40 kb in length. Major gene loss that occurred among members of the order *Lactobacillales* indicates early adaptation to nutritionally rich environments (12). One deletion of interest within contig 11, which mapped within the 448212 to 476326 region of CNRZ 32, shows the lack of a cell wall rhamnose containing a polysaccharide cluster. Additionally, in the same deletion we noticed the lack of a glicosyltransferase and an alpha-d-GlcNac-alpha-1,2-l-rhamnosyltransferase, both possibly involved in the side chain formation of rhamnose-glucose cell wall polysaccharides. The lack of these genes would probably produce variability in the cell wall polysaccharide architecture. It was found that changes in autolytic properties observed in different strains of *L. helveticus* were due to differences in the chemical structure of their cell wall polysaccharides (13). Autolysis of the starter’s strains, as those belonging to the *L. helveticus* species, remains of considerable interest regarding their use in dairy fermentation, particularly in the manufacture of cheeses.

### Accession number(s).

This whole-genome shotgun project has been deposited at DDBJ/EMBL/GenBank under the accession number PJRL00000000. The version described in this paper is version PJRL01000000.

## References

[1] Taverniti V, Guglielmetti S 2012 Health-promoting properties of *Lactobacillus helveticus*. Front Microbiol 3:392. doi:10.3389/fmicb.2012.00392. 23181058 PMC3500876

[2] Johnson-Henry KC, Hagen KE, Gordonpour M, Tompkins TA, Sherman PM 2007 Surface-layer protein extracts from *Lactobacillus helveticus* inhibit enterohaemorrhagic *Escherichia coli* O157:H7 adhesion to epithelial cells. Cell Microbiol 9:356–367. doi:10.1111/j.1462-5822.2006.00791.x. 16925785

[3] Taverniti V, Stuknyte M, Minuzzo M, Arioli S, De Noni I, Scabiosi C, Cordova ZM, Junttila I, Hämäläinen S, Turpeinen H, Mora D, Karp M, Pesu M, Guglielmetti S 2013 S-layer protein mediates the stimulatory effect of *Lactobacillus helveticus* MIMLh5 on innate immunity. Appl Environ Microbiol 79:1221–1231. doi:10.1128/AEM.03056-12. 23220964 PMC3568609

[4] Meng J, Zhu X, Gao SM, Zhang QX, Sun Z, Lu RR 2014 Characterization of surface layer proteins and its role in probiotic properties of three *Lactobacillus* strains. Int J Biol Macromol 65:110–114. doi:10.1016/j.ijbiomac.2014.01.024. 24444879

[5] Skrzypczak K, Gustaw W, Waśko A 2015 Health-promoting properties exhibited by *Lactobacillus helveticus* strains. Acta Biochim Pol 62:713–720. doi:10.18388/abp.2015_1116. 26601325

[6] Prado Acosta M, Ruzal SM, Cordo SM 2016 S-layer proteins from *Lactobacillus* sp. inhibit bacterial infection by blockage of DC-SIGN cell receptor. Int J Biol Macromol 92:998–1005. doi:10.1016/j.ijbiomac.2016.07.096. 27498415

[7] Bankevich A, Nurk S, Antipov D, Gurevich AA, Dvorkin M, Kulikov AS, Lesin VM, Nikolenko SI, Pham S, Prjibelski AD, Pyshkin AV, Sirotkin AV, Vyahhi N, Tesler G, Alekseyev MA, Pevzner PA 2012 SPAdes: a new genome assembly algorithm and its applications to single-cell sequencing. J Comput Biol 19:455–477. doi:10.1089/cmb.2012.0021. 22506599 PMC3342519

[8] Boetzer M, Henkel CV, Jansen HJ, Butler D, Pirovano W 2011 Scaffolding pre-assembled contigs using SSPACE. Bioinformatics 27:578–579. doi:10.1093/bioinformatics/btq683. 21149342

[9] Sosa EJ, Burguener G, Lanzarotti E, Defelipe L, Radusky L, Pardo AM, Marti M, Turjanski AG, Do Porto DF 2018 Target-Pathogen: a structural bioinformatic approach to prioritize drug targets in pathogens. Nucleic Acids Res 46:D413–D418. doi:10.1093/nar/gkx1015. 29106651 PMC5753371

[10] Aziz RK, Bartels D, Best AA, DeJongh M, Disz T, Edwards RA, Formsma K, Gerdes S, Glass EM, Kubal M, Meyer F, Olsen GJ, Olson R, Osterman AL, Overbeek RA, McNeil LK, Paarmann D, Paczian T, Parrello B, Pusch GD, Reich C, Stevens R, Vassieva O, Vonstein V, Wilke A, Zagnitko O 2008 The RAST server: Rapid Annotations using Subsystems Technology. BMC Genomics 9:75. doi:10.1186/1471-2164-9-75. 18261238 PMC2265698

[11] Callanan M, Kaleta P, O’Callaghan J, O’Sullivan O, Jordan K, McAuliffe O, Sangrador-Vegas A, Slattery L, Fitzgerald GF, Beresford T, Ross RP 2008 Genome sequence of *Lactobacillus helveticus*, an organism distinguished by selective gene loss and insertion sequence element expansion. J Bacteriol 190:727–735. doi:10.1128/JB.01295-07. 17993529 PMC2223680

[12] Makarova KS, Slesarev A, Wolf Y, Sorokin A, Mirkin B, Koonin EV, Pavlov A, Pavlova N, Karamychev V, Polouchine N, Shakhova V, Grigoriev I, Lou Y, Rohksar D, Lucas S, Huang K, Goodstein DM, Hawkins T, Plengvidhya V, Welker DL, Hughes JE, Goh Y, Benson A, Baldwin KA, Lee JH, Díaz-Muñiz I, Dosti B, Smeianov VV, Wechter W, Barabote R, Lorca GL, Altermann E, Barrangou R, Ganesan B, Xie Y, Rawsthorne H, Tamir D, Parker C, Breidt F, Broadbent JR, Hutkins R, O’Sullivan D, Steele JL, Unlu G, Saier MH, Klaenhammer TR, Richardson P, Kozyavkin S, Weimer BC, Mills DA 2006 Comparative genomics of the lactic acid bacteria. Proc Natl Acad Sci U S A 103:15611–15616. doi:10.1073/pnas.0607117103. 17030793 PMC1622870

[13] Vinogradov E, Valence F, Maes E, Jebava I, Chuat V, Lortal S, Grard T, Guerardel Y, Sadovskaya I 2013 Structural studies of the cell wall polysaccharides from three strains of *Lactobacillus helveticus* with different autolytic properties: DPC4571, BROI, and LH1. Carbohydr Res 379:7–12. doi:10.1016/j.carres.2013.05.020. 23831635

